# Effect of Alpha-Linolenic Acid Supplementation on Cardiovascular Disease Risk Profile in Individuals with Obesity or Overweight: A Systematic Review and Meta-Analysis of Randomized Controlled Trials

**DOI:** 10.1016/j.advnut.2023.09.010

**Published:** 2023-09-29

**Authors:** Shiyu Yin, Hai Xu, Jiayue Xia, Yifei Lu, Dengfeng Xu, Jihan Sun, Yuanyuan Wang, Wang Liao, Guiju Sun

**Affiliations:** 1 Department of Nutrition and Food Hygiene, Key Laboratory of Environmental Medicine and Engineering of Ministry of Education, School of Public Health, Southeast University, Nanjing, China; 2 Department of Food Processing and Safety, College of Biology and Food Engineering, Chongqing Three Gorges University, Chongqing, China; 3 China-DRIs Expert Committee on Macronutrients, Chinese Nutrition Society, Beijing, China

**Keywords:** alpha-linolenic acid, cardiovascular disease risk, overweight, obesity, inflammation, blood pressure, blood lipid

## Abstract

Overweight and obesity are highly prevalent worldwide and are associated with cardiovascular disease (CVD) risk factors, including systematic inflammation, dyslipidemia, and hypertension. Alpha-linolenic acid (ALA) is a plant-based essential polyunsaturated fatty acid associated with reduced CVD risks. This systematic review and meta-analysis aimed to investigate the effects of supplementation with ALA compared with the placebo on CVD risk factors in people with obesity or overweight (International Prospective Register of Systematic Reviews Registration No. CRD42023429563). This review included studies with adults using oral supplementation or food or combined interventions containing vegetable sources of ALA. All studies were randomly assigned trials with parallel or crossover designs. The Cochrane Collaboration tool was used for assessing the risk of bias (Version 1). PubMed, Web of Science, Embase, and Cochrane library databases were searched from inception to April 2023. Nineteen eligible randomized controlled trials, including 1183 participants, were included in the meta-analysis. Compared with placebo, dietary ALA supplementation significantly reduced C-reactive protein concentration (standardized mean difference [SMD] = –0.38 mg/L; 95% confidence interval [CI]: –0.72, –0.04), tumor necrosis factor-α concentration (SMD = –0.45 pg/mL; 95% CI: –0.73, –0.17), triglyceride in serum (SMD = −4.41 mg/dL; 95% CI: –5.99, –2.82), and systolic blood pressure (SMD = –0.37 mm Hg; 95% CI: –0.66, –0.08); but led to a significant increase in low-density lipoprotein cholesterol concentrations (SMD = 1.32 mg/dL; 95% CI: 0.05, 2.59). ALA supplementation had no significant effect on interleukin-6, diastolic blood pressure, total cholesterol, or high-density lipoprotein cholesterol (all *P* ≥ 0.05). Subgroup analysis revealed that ALA supplementation at a dose of ≥3 g/d from flaxseed and flaxseed oil had a more prominent effect on improving CVD risk profiles, particularly where the intervention duration was ≥12 wk and where the baseline CVD profile was poor.

**Statements of Significance**

To our knowledge, this is the first comprehensive meta-analysis to summarize current evidence on the effect of ALA supplementation on cardiovascular disease risk factors in people with obesity or overweight.

## Introduction

Overweight and obesity are global health epidemics. According to the WHO estimates, >50% of the world’s adult population is overweight or obese [1,2]. WHO defines overweight as a BMI (kg/m^2^) of ≥25 and <30 and obesity as a BMI of ≥30 kg/m^2^ [3]. Overweight and obesity are consistently and strongly associated cardiovascular disease (CVD) risk factors, including a hypertensive state, dyslipidemia characterized by elevated serum concentrations of triglycerides (TGs), elevated serum concentrations of total cholesterol (TC), increased serum concentrations of LDL cholesterol, and decreased serum concentrations of HDL cholesterol, and chronic low-grade inflammation [[4], [5], [6]].

As 1 of the leading causes of mortality and morbidity in the world, CVD accounted for 32% of total global death in 2019 [7,8]. Strong clinical and epidemiologic evidence suggests that obesity and overweight are linked to a wide variety of CVDs, including heart failure, coronary artery disease, atrial fibrillation, ventricular arrhythmias, cerebrovascular disease, and sudden cardiac death [[9], [10], [11], [12]]. Obesity and overweight contribute to CVD morbidity and mortality directly through the proinflammatory adipokine effect on vascular homeostasis and indirectly mediated by coexisting CVD risk factors, including hypertension and dyslipidemia [13]. The pathophysiologic mechanism of CVD development in obesity or overweight is linked to hemodynamic effects and cardiac adaptations [13]. Lifestyle modifications, such as exercise, a healthy diet, and avoiding smoking and alcohol could reduce the risk of CVD [[14], [15], [16], [17]].

The favorable effects of omega-3 PUFAs (n–3 PUFA), particularly EPA (20:5 n–3) and DHA (22:6 n–3) for cardiometabolic health have been well demonstrated [18]. EPA and DHA are able to reduce TG and TC either in combination or as single components. A recent science advisory from the American Heart Association has addressed the use of n–3 PUFA supplementation to reduce CVD risk [19]. However, dietary habits, issues of palatability, compromised fish supplies, methyl mercury contamination, and concerns about sustainability make it difficult for most people to maintain an adequate n–3 PUFA status. Very low blood concentrations of EPA and DHA were observed in North America, Central and South America, Europe, the Middle East, Southeast Asia, and Africa [20].

This has urged a search for alternative n–3 PUFA sources. The n–3 PUFA precursor, α-linolenic acid (ALA; 18:3 n–3), can be converted to EPA and DHA via desaturation and elongation in mammals [21]. Through the action of delta 6-desaturase and delta 5-desaturase, ALA is desaturated to stearidonic acid and EPA consecutively in the liver endoplasmic reticulum. EPA is then extended to tetracosahexaenoic acid by elongase-2 and transferred to peroxisomes in the liver to form DHA via β-oxidation [22]. However, the metabolic conversion process in the human body is relatively limited. The conversion rate of ALA to EPA is estimated to be <8%, and ALA to DHA is <4%. Moreover, the conversion rate varied across the population of different ages, sex, or disease [23]. The obesity-associated changes in desaturase and elongase activities could influence the metabolic conversion of ALA. In subjects with obesity or overweight, the delta 6-desaturase activity increases, whereas delta 5-desaturase decreases in plasma, adipose tissue, and erythrocytes [24,25]. Therefore, the conversion rate of ALA to EPA/DHA is unpredictable.

ALA is abundant in flaxseed, walnuts, and perilla and in other foods at lower concentrations [9,10], which is more affordable and has greater global availability. Increasing ALA consumption could reduce the requirement for fish and help preserve the fish supply. Dietary ALA intake is associated with a reduced risk of CVD mortality and all-cause mortality [26]. In 2021, a meta-analysis of 47 randomized controlled trials (RCTs) by Yue et al. [27] found that dietary intake of ALA improves blood lipid profiles by reducing the concentrations of TG, TC, LDL, and VLDL cholesterol, demonstrating that increasing ALA intake could potentially prevent the risk of CVDs.

Myriad scientific research has investigated the effect of ALA supplementation on CVD risk factors in populations of different health conditions; however, the findings of these studies remain controversial [[28], [29], [30], [31], [32]]. Kamoun et al. [32] found that 6-wk moderate walnut supplementation (15 g/d) improved lipid profile and systematic inflammation in trained men aged ≥65 y, whereas a meta-analysis by Su et al. [29] found no beneficial effect of ALA supplementation on reducing inflammatory markers. Another meta-analysis of 20 RCTs showed that flaxseed and its derivatives did not change circulating C-reactive protein (CRP) despite populations with obesity [30]. These conflicting results regarding various CVD risk factors indicate that the effect of dietary ALA on CVD risk might depend on the health status of the population. Thus, the current meta-analysis was performed to summarize the available evidence regarding the effects of dietary ALA supplementation inflammatory markers in serum (CRP, TNF-α, and IL-6), blood pressure ([BP], including systolic BP [SBP] and diastolic BP [DBP]), and lipid profile (TG, TC, LDL cholesterol, and HDL cholesterol in serum) in individuals with obesity or overweight as compared with the placebo. This will provide health professionals and policymakers with updated evidence to make more specific dietary recommendations for people with obesity or overweight.

## Methods

### Protocol and registration

This systematic review and meta-analysis were prospectively registered in the search strategy (PROSPERO Registration No. CRD42023429563). The review protocol can be accessed at =https://www.crd.york.ac.uk/prospero/display_record.php?ID=CRD42023429563) and was strictly followed when conducting. It was reported in line with the PRISMA statement (Supplementary Table 1) [33]. All studies included in this review were confirmed to be free of ethical/moral concerns. Therefore, the need for ethics committee consent was waived.

### Eligibility criteria

Studies were included if they met the following inclusion criteria: *1*) RCT with parallel or crossover design; *2*) the intervention group used dietary supplementation with ALA (including using flaxseed, chia seed, linseed, canola, rapeseed, walnut, perilla, and their derivates as sources of ALA); *3*) the control group used iso-caloric food as a placebo; *4*) participants in both the intervention and control groups had a BMI of ≥25; *5*) supplementations should not contain fatty acids that have been reported to influence inflammatory response, e.g., γ-linolenic acid, conjugated linoleic acid, and long-chain PUFA, including arachidonic acid and EPA; *6*) intervention duration lasted no <4 wk [34]; *7*) investigated the effects of ALA supplementation of any source/dose on CVD risk factors, including inflammatory markers (CRP, TNF-α, and IL-6), BP (SBP and DBP), and lipid profile (TG, TC, LDL cholesterol, and HDL cholesterol); and *8*) provided enough data regarding the outcome of interest to extract or calculate the mean difference and SD from the baseline to the end point.

Exclusion criteria were studies with no comparator; participants were pediatric patients or pregnant women and nursing mothers and enteral or parenteral feeding, or in vitro, in vivo, and ex vivo studies.

### Study identification and selection

Potential literature was searched in PubMed, Web of Science, Embase, and Cochrane library databases from journal inception dates to 25 April 2023. Terms, such as “Alpha-linolenic acid,” “Omega-3 fatty acid,” “flaxseed oil,” “chia seed oil,” “linseed oil,” “canola oil,” “rapeseed oil,” “walnut oil,” and “perilla oil,” “overweight,” “obesity” were used as keywords in the search. The complete search strategy and results in all 4 databases are provided in Supplementary Table 2. Bibliographies of retrieved articles were manually searched to make sure no relevant studies were missed. Potential studies were screened independently by 2 reviewers (SY and HX). Any discrepancies were resolved by discussion with a third reviewer (JX) until consensus was reached.

### Data extraction and quality assessment

Data extraction and quality assessment were conducted independently by 2 investigators. Disagreements between the 2 were resolved by discussion with a third investigator until a consensus was reached. The following characteristics were extracted: the surname of the first author, year of publication, region, smoking status, sample size, study duration, study design, ALA source and dose, and outcomes evaluated.

The quality of selected studies was assessed according to the Cochrane Collaboration’s tool for assessing the risk of bias [35]. The assessment is composed of 7 items, namely random-sequence generation, allocation concealment, blinding of participants and personnel, blinding of outcome “Low,” “High,” or “Unclear.” The overall quality of the individual studies was determined as good quality if the study scored “low risk” for all items, fair quality if the study scores unclear risk for 1 item or more, or poor quality if the study scores high risk for 1 item or more.

### Statistical analysis

Comprehensive Meta-Analysis version 3.3 (Biostat, Inc.) was used for statistical analysis. Standardized mean difference (SMD) with a corresponding 95% confidence interval (CI) was used to report effect sizes in meta-analysis to accommodate the differences in the measurements that varied across the included studies. Forest plots were generated for each outcome [36]. The mean difference between baseline and SD in both intervention and control groups was extracted or calculated in each trial. The mean difference can be obtained by subtracting the mean of the baseline from that of the end point. The SD was calculated based on the SE, 95% CI or median, and interquartile range (IQR) when necessary. SD of net change was calculated with the following equation: SD = square root [(SD _pretreatment_)^2^ + (SD _posttreatment_) ^2^ − (2R × SD _pretreatment_ × SD _posttreatment_)^2^], where the value of *R* was assumed to be 0.8 [37]. When median and IQR are only provided, the mean could be approximated as the median, and SD can be calculated as IQR/1.35 [38]. For crossover trials, we used an *R* of 0.5 to calculate SD in pairwise analysis [39]. We included each effect size when several intervention arms were used in studies and divided the corresponding sample size accordingly to minimize a unit of analysis error [36]. If the end points were measured multiple times during the intervention, only the last 1 was included in the analysis. *I*^*2*^ statistic and Cochrane’s Q test were used to assess the heterogeneity between studies. *I*^2^ values of 25%, 50%, and 75% were considered low, moderate, and high levels of heterogeneity, respectively. The random-effects model was used for high heterogeneity; otherwise, the fixed-effects model was used [40]. Subgroup analysis was performed to identify the potential source of heterogeneity based on the following categories: study duration (<12 or ≥12 wk), ALA dose difference between intervention and control group (<3 or ≥3 g), ALA source (flaxseed and its derivatives, walnuts, or others), baseline BMI (<30 or ≥30), and baseline levels of inflammatory markers, BP, blood lipid, and study quality. Publication bias was assessed using visual inspection of funnel plots and statistically using Begg’s and Egger’s regression tests (significant when *P* value <0.05). If asymmetry exists by visual inspection of the funnel plot, a nonparametric “trim and fill” analysis was performed to estimate potentially missing studies and to adjust results. Sensitivity analysis with the leave-one-out method was employed to evaluate whether the overall effect size changes if 1 single meta-analysis is omitted.

## Results

### Study selection

**FIGURE 1:**
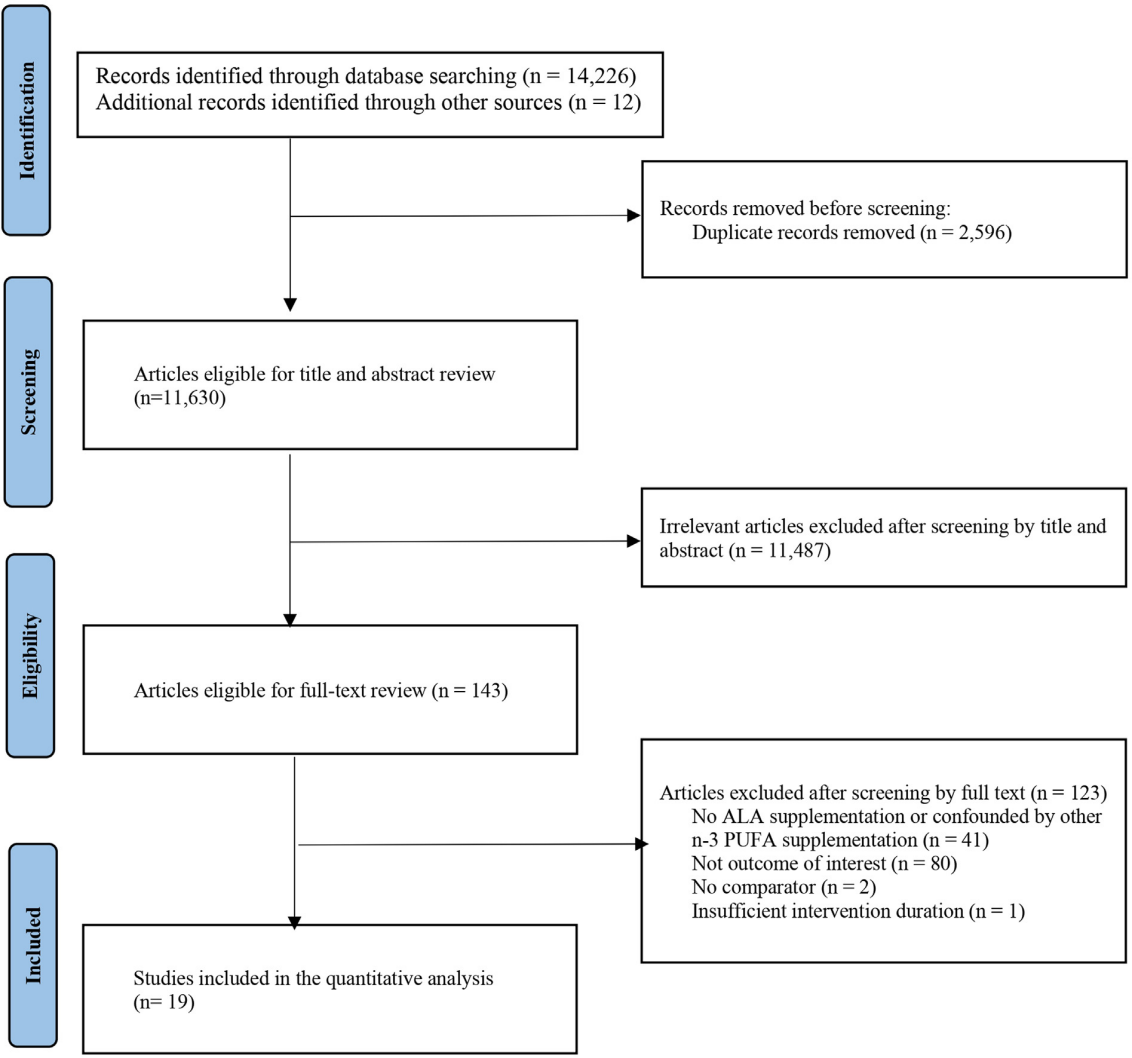
The flow diagram of eligible studies. ALA, alpha-linolenic acid.

As shown in Figure 1, the primary search yielded 14,238 records. After the removal of 2596 duplicates, 11,630 records remained. After screening by title and abstract, 11,487 irrelevant studies were discarded because of study design, study subject, type of intervention, and evaluated outcome. Of 143 articles eligible for full-text evaluation, 123 were removed for the following reasons: 46 did not implement ALA intervention, 41 did not use ALA supplementation or confounded with other n–3 supplements, 80 lacked data on outcomes, 2 without suitable comparator, and 1 without adequate intervention duration. Finally, 19 studies involving 1183 participants were eligible for the current meta-analysis.

### Characteristics of the included studies

The characteristics of each included study are described in Table 1. Of 19 included studies published between 2007 and 2022, 5 were conducted in the United States [[41], [42], [43], [44], [45]], 4 in Brazil [[46], [47], [48], [49]], 4 in Iran [[50], [51], [52], [53]], 2 in Germany [54,55], 1 in Australia [56], 1 in Korea [57], 1 in Sweden [58], and 1 in Netherland [59].

**TABLE 1:** Characteristics of the 19 included studies

Author, y	Region	Study design	Duration (wk)	No. (I/C)	ALA source	ALA dose (g/d)	Control	Smoking status	Outcomes
Ahmadniay et al., 2021 [50]	Iran	P	12	52 (29/23)	Flaxseed (brown, milled)	5.82	Milled rice	Nonsmoking	TC, TG, LDL cholesterol, HDL cholesterol
Baxheinrich et al., 2012 [55]	Germany	P	26	81 (40/41)	Rapeseed oil	2.235	Olive oil	Nonsmoking	SBP, DBP
de Oliveira et al., 2017 [46]	Brazil	P	12	52 (26/26)	Flaxseed oil	16.02	Sunflower oil	Nonsmoking	CRP
Egert et al., 2014 [60]	Germany	P	26	81 (40/41)	Rapeseed oil	3.4	Olive oil	Nonsmoking	CRP, TNF, IL-6, SBP, DBP
Eriksen et al., 2020 [58]	Sweden	CO	8	78 (39/39)	Lignan	0.28	Wheat	Nonsmoking	TC, TG, LDL cholesterol, HDL cholesterol, SBP, DBP
Faintuch et al., 2011 [47]	Brazil	CO	12	18 (10/8)	Flaxseed (powder)	10	Cassava	Nonsmoking	CRP, SBP, DBP
Hajiahmadi et al., 2020 [51]	Iran	P	14	36 (18/18)	Flaxseed oil	2	Oral paraffin	Nonsmoking	TNF
Hutchins et al., 2013 [41]	USA	CO	12	25	Flaxseed oil	13.884	Control diet	Nonsmoking	CRP, IL-6
Hwang et al., 2019 [57]	Korea	CO	16	84 (43/41)	Walnut	4.086	White bread	Nonsmoking	TC, TG, LDL cholesterol, HDL cholesterol, SBP, DBP
Jamilian et al., 2019 [53]	Iran	P	12	40 (20/20)	Flaxseed oil	1	Placebo	Nonsmoking	CRP, TC, TG, LDL cholesterol, HDL cholesterol
Joris et al., 2020 [61]	Netherland	P	12	59 (29/30)	Flaxseed oil	4.7	Sunflower oil	NR	CRP, TNF, IL-6, TC, TG, LDL cholesterol, HDL cholesterol
Katz et al., 2012 [42]	USA	CO	8	46 (23/23)	Walnut	5.0848	Control diet	Nonsmoking	TC, TG, LDL cholesterol, HDL cholesterol, SBP, DBP
Kruse et al., 2020 [48]	Brazil	P	8	26 (15/11)	Rapeseed oil	3.725	Olive oil	Nonsmoking	CRP
Machado et al., 2015^a^ [49]	Brazil	P	11	24 (12/12)	Flaxseed (brown)	5.432	Wheat bran	NR	CRP, TNF, IL-6, TC, TG, LDL cholesterol, HDL cholesterol
Machado et al., 2015^b^ [49]	Brazil	P	11	26 (14/12)	Flaxseed (golden)	5.432	Wheat bran	NR	CRP, TNF, IL-6, TC, TG, LDL cholesterol, HDL cholesterol
Ndanuko et al., 2018 [56]	Australia	P	12	144 (82/62)	Walnut	2.724	Wheat bran	NR	SBP, DBP
Nelson et al., 2007 [44]	USA	P	8	51 (27/24)	Flaxseed oil	11	Control diet	Nonsmoking	CRP, TNF, IL-6
Nieman et al., 2012^c^ [44]	USA	P	10	42 (16/26)	Chia seed (whole)	4.45	Poppy seed	Nonsmoking	CRP, TNF, IL-6, SBP, TC
Nieman et al., 2012^d^ [44]	USA	P	10	40 (14/26)	Chia seed (milled)	4.45	Poppy seed	Nonsmoking	CRP, TNF, IL-6, SBP, TC
Rock et al., 2017 [45]	USA	P	12	110 (51/49)	Walnut	3.75	Control diet	Nonsmoking	TC, TG, LDL cholesterol, HDL cholesterol, SBP, DBP
Shareghfarid et al., 2022 [52]	Iran	P	14	68 (32/36)	Flaxseed oil	2	Placebo	Nonsmoking	TNF

Abbreviations: ALA, alpha-linolenic acid; C, control; CO, crossover; CRP, C-reactive protein; DBP, diastolic blood pressure; I, intervention; NR, not reported; P, parallel; SBP, systolic blood pressure; TC, total cholesterol; TG, triglyceride.

a Intervention arm used brown flaxseed as ALA source.

b Intervention arm used brown flaxseed as ALA source.

c Intervention arm used whole chia seed as ALA source.

d Intervention arm used milled chia seed as ALA source.

Twelve trials investigated inflammatory markers as an outcome [41,43,44,[46], [47], [48], [49],[51], [52], [53],^59^,^60^], 9 assessed BP [42,44,45,47,[55], [56], [57], [58],^60^], and 9 assessed blood lipid profile [42,44,45,49,50,53,[57], [58], [59]]. All the 19 selected studies had a proper controlled design, with the only difference between the intervention and control groups being the dietary ALA intervention. Intervention duration ranged from 8 to 26 wk, with more than half of the trials conducted for ≥12 wk. The control group consumed food low in ALA as a placebo, such as olive oil [55,60], sunflower oil [46,61], wheat bran [49,56,58], etc. Two studies did not specify the type of placebo [52,53]. The source and doses of ALA supplementation used in the intervention groups varied across the 19 included studies. Regarding the source of ALA, 3 used flaxseed [47,49,50], 7 used flaxseed oil [41,43,46,[51], [52], [53],^61^], 3 used rapeseed oil [48,55,60], 4 used walnut [42,45,56,57], 1 used lignan [58], and 1 used chia seed [44]. Doses of ALA supplementation ranged from 0.28 to 16.02 g/d.

### Quality assessment

The risk of bias in each trial was evaluated in Supplementary Table 3. Overall, 5 trials were assessed as good quality, 7 as fair quality, and 8 as poor quality. Regarding random-sequence generation, 12 studies had a low risk of bias [42,43,45,[48], [49], [50],^52^,^53^,[56], [57], [58],^61^], and the rest showed unclear risk of bias [46,55,60]. In terms of allocation concealment, 8 trials showed a low risk of bias, 6 had a high risk of bias, and the rest showed unclear risk of bias. Eight trials had reported blinding of participants had personnel, and 12 trials reported blinding of outcome assessments. All but 2 trials had a low risk of outcome reporting, whereas all trials had a low risk of bias in terms of other potential threats to validity.

### Meta-analysis

#### Effect of ALA on inflammatory markers

##### Effect of ALA on serum CRP concentrations

**FIGURE 2:**
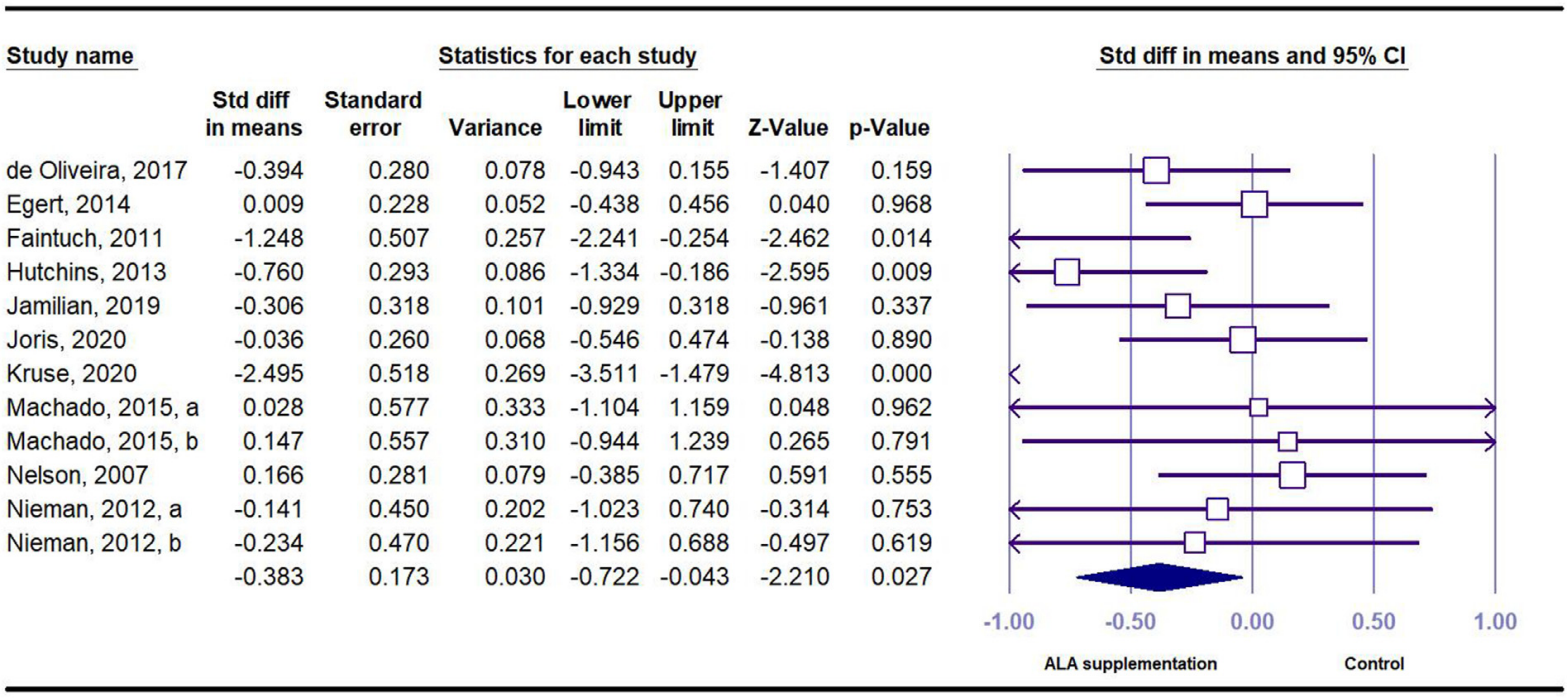
Forest plot of standard mean difference and 95% confidence interval for the effect of dietary ALA supplementation on C-reactive protein in people with obesity or overweight. ALA, alpha-linolenic acid.

A total of 12 effect sizes (*n* = 220 intervention and *n* = 221 control) were obtained from 10 RCTs that investigated the effect of ALA on CRP in serum. The results showed that compared with placebo, dietary intake of ALA had a significant reduction in serum CRP concentration (SMD = –0.38 mg/L; 95% CI: –0.72, –0.04) with a moderate level of between-study heterogeneity found (*I*^*2*^ = 64.20, *P* < 0.001) (Figure 2). In the subgroup analysis, the effects remained significant only in studies with longer duration (≥12 wk), used flaxseed and its derivatives as ALA source, or used higher doses of ALA (≥3 g), included participants with higher baseline BMI (≥30 kg/m^2^), or included participant with higher serum CRP concentration at baseline (≥3.0 mg/L) (Supplementary Table 4).

##### Effect of ALA on serum TNF-α concentrations

**FIGURE 3:**
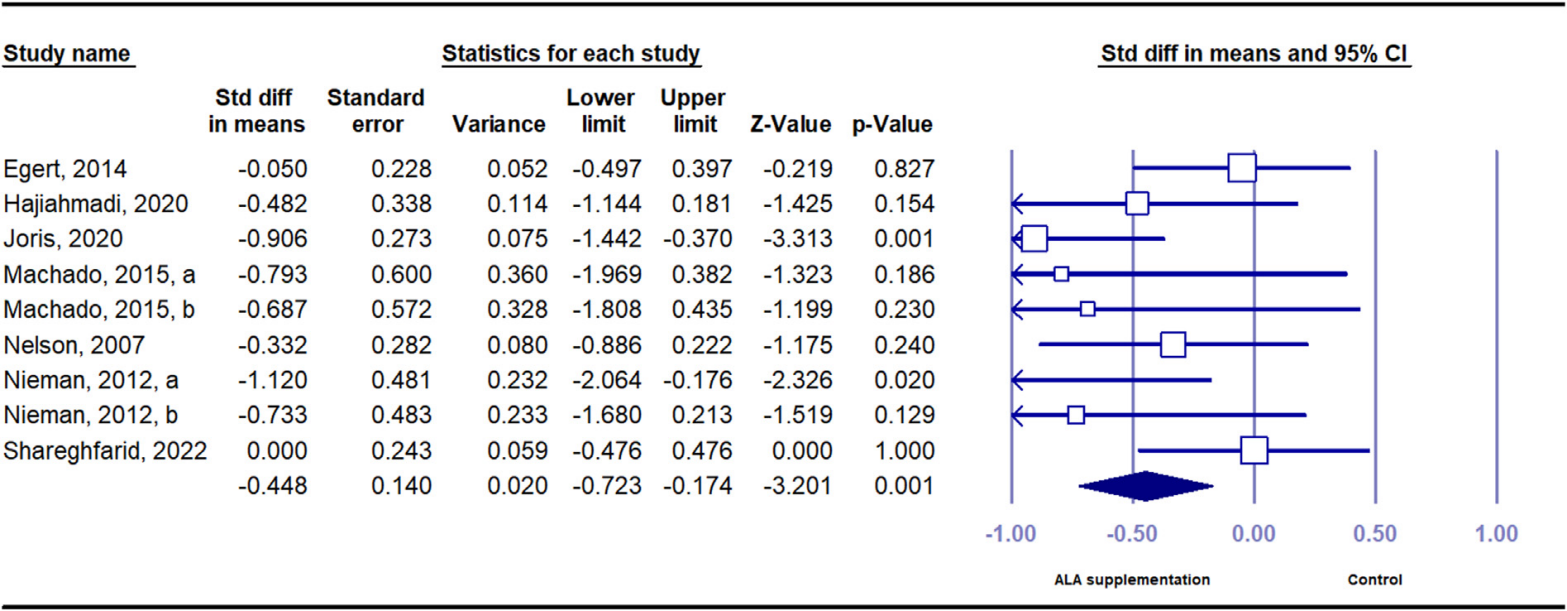
Forest plot of standard mean difference and 95% confidence interval for the effect of dietary ALA supplementation on TNF-α in people with obesity or overweight. ALA, alpha-linolenic acid.

TNF-α was evaluated in 7 studies with 9 effect sizes (*n* = 172 intervention and *n* = 185 control). Pooled results suggested that compared with placebo, dietary ALA significantly decreased serum TNF-α concentration (SMD = –0.45 pg/mL, 95% CI: –0.72, –0.17) with a low level of between-study heterogeneity (*I*^*2*^ = 33.45, *P* = 0.15) (Figure 3). Subgroup analysis showed that the effect of ALA on serum TNF-α remained significant in studies with longer duration (≥12 wk), those included participants with higher serum TNF-α concentration at baseline (≥2.2 pg/mL), included participants with higher baseline BMI (≥30), those used flaxseed and its derivatives as ALA source, used higher doses of ALA (≥3 g), or those of fair quality (Supplementary Table 4).

##### Effect of ALA on serum IL-6 concentrations

**FIGURE 4:**
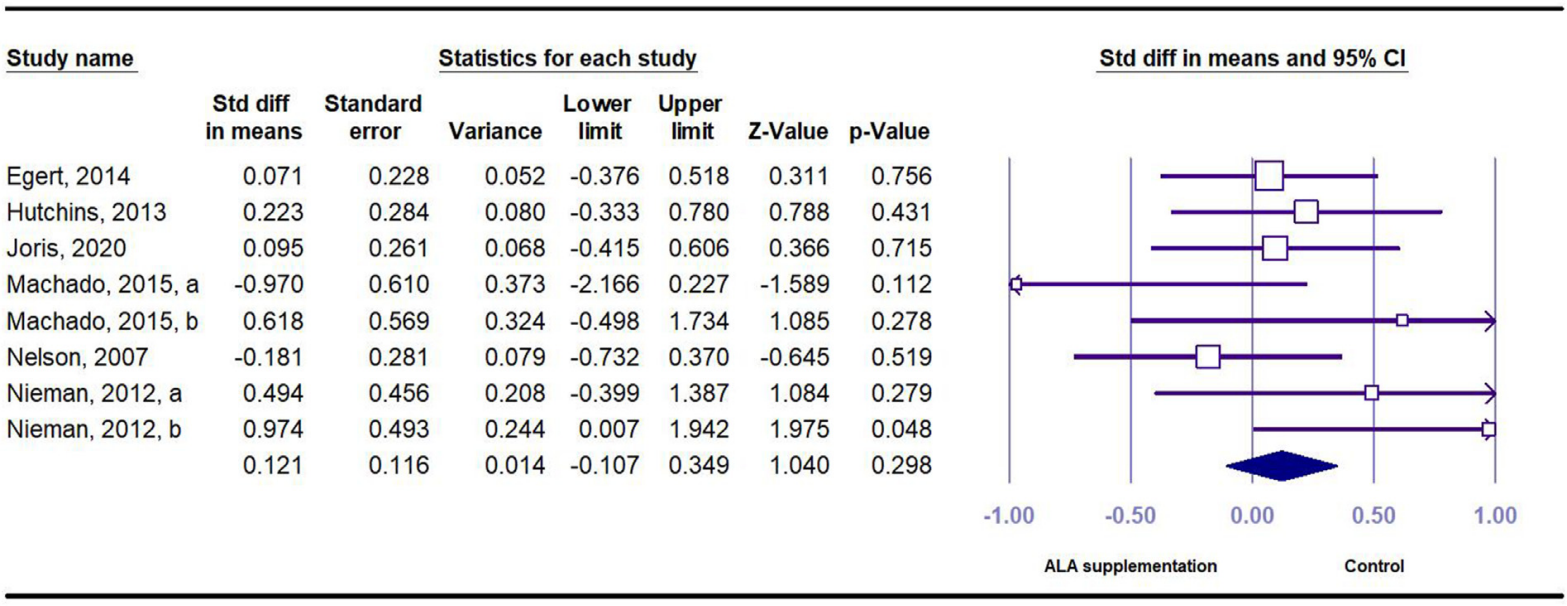
Forest plot of standard mean difference and 95% confidence interval for the effect of dietary ALA supplementation on IL-6 in people with obesity or overweight. ALA, alpha-linolenic acid.

Six RCTs provided 8 effect sizes (*n* = 147 intervention and *n* = 156 control) and reported IL-6 as an outcome measure. These studies suggested no significant association between dietary ALA and serum IL-6 concentration (SMD = 0.12 pg/mL; 95% CI: –0.14, 0.40; *I*^*2*^ = 21.87) (Figure 4). Subgroup analysis revealed that the effect remained nonsignificant across all subgroups (Supplementary Table 4).

#### Effect of ALA on BP

##### Effect of ALA on SBP

**FIGURE 5:**
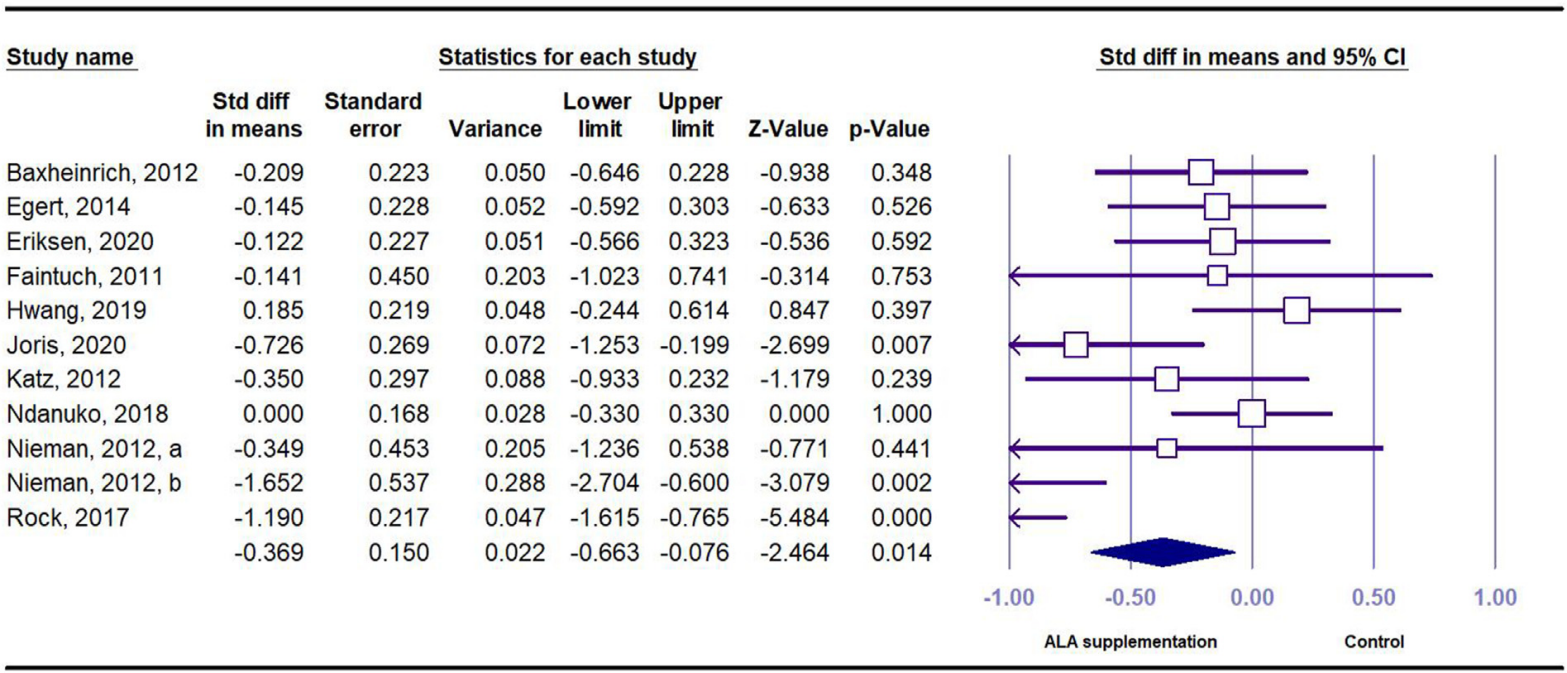
Forest plot of standard mean difference and 95% confidence interval for the effect of dietary ALA supplementation on systolic blood pressure in people with obesity or overweight. ALA, alpha-linolenic acid.

Pooled data from 10 studies indicated that compared with placebo, dietary intake of ALA significantly decreased SBP (SMD = –0.37 mm Hg; 95% CI: –0.66, –0.08); however, substantial between-study heterogeneity was found (*I*^*2*^ = 71.58%, *P* < 0.001) (Figure 5). In the subgroup analysis, the significant effects were only observed in studies with longer duration (≥12 wk), used flaxseed and its derivatives as ALA source, or used higher doses of ALA (≥3 g), included participants with higher baseline BMI (≥30 kg/m^2^), included participant with hypertension at baseline (SBP ≥ 130 or DBP ≥ 80 mm Hg), or studies of fair quality (Supplementary Table 4).

##### Effect of ALA on DBP

**FIGURE 6:**
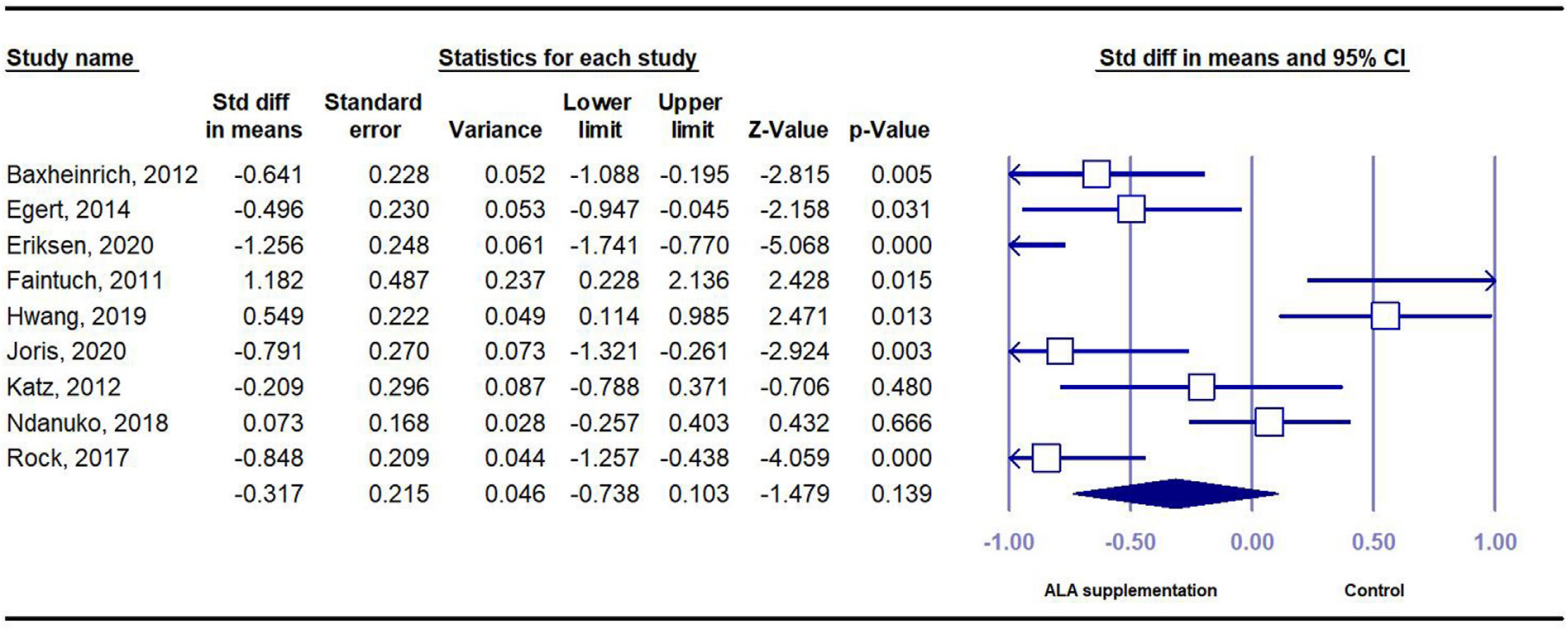
Forest plot of standard mean difference and 95% confidence interval for the effect of dietary ALA supplementation on diastolic blood pressure in people with obesity or overweight. ALA, alpha-linolenic acid.

In total, 9 RCTs (*n* = 357 intervention and *n* = 332 control) investigated the effect of ALA on DBP. These studies suggested no significant change of DBP following ALA intervention (SMD = –0.32; 95% CI: –1.26, 0.54) with substantial between-study heterogeneity (*I*^*2*^ = 85.85; *P* = 0.003) (Figure 6). Subgroup analysis showed that the ALA intake significantly reduced DBP in studies that used ALA sources other than flaxseed and walnut or those of fair quality (Supplementary Table 4).

#### Effect of ALA on lipid profile

##### Effect of ALA on TG concentration in serum

Nine RCTs provided 10 effect sizes (*n* = 258 intervention and *n* = 247 control) and reported TG in serum as an outcome measure. Pooled results demonstrated that ALA supplementation significantly reduced serum TG concentration compared with the control group (SMD = –4.41 mg/dL; 95% CI: –5.99, –2.82); however, the between-study heterogeneity was substantial (*I*^*2*^ = 83.08; *P* = 0.003) (Supplementary Figure 1). In subgroup analysis, the effect was no longer significant in studies with short duration (<12 wk), used walnuts as ALA source, or used lower doses of ALA supplementation (<3 g/d), whereas the rest remained significant. (Supplementary Table 4).

##### Effect of ALA on TC concentration in serum

Meta-analysis of data from 10 studies with 12 effect sizes (*n* = 273 intervention and *n* = 271 control) showed that ALA intake has no effect on serum TC concentration (SMD = 0.60; 95% CI: –0.33, 1.53; *I*^*2*^ = 95.16) (Supplementary Figure 2). Subgroup analysis showed that the effect of ALA on TC was significant in studies that used walnuts as ALA sources or those of good quality (Supplementary Table 4).

##### Effect of ALA on LDL cholesterol concentration in serum

Pooled results from 8 RCTs providing 10 effect sizes (*n* = 258 intervention and *n* = 245 control) revealed that ALA supplementation led to an increase in serum LDL cholesterol concentrations (SMD = 1.32; 95% CI: 0.05, 22.59) with substantial between-study heterogeneity (*I*^*2*^ = 96.76; *P* < 0.001) (Supplementary Figure 3). In subgroup analysis, the effect only remained significant in studies that enrolled participants with normal blood lipid profiles at baseline, included participants with high baseline BMI (≥30 kg/m^2^), used flaxseed and its derivatives as an ALA source, or of poor quality (Supplementary Table 4).

##### Effect of ALA on HDL cholesterol concentration in serum

HDL cholesterol concentration was evaluated in 8 studies with 10 effect sizes (*n* = 258 intervention and *n* = 245 control). Pooled results suggested dietary ALA had no effect on HDL cholesterol concentration (SMD = –0.50; 95% CI: –1.456, 0.45; *I*^*2*^ = 95.03) (Supplementary Figure 4). However, subgroup analysis showed that ALA supplementation had a significant effect on reducing HDL cholesterol in studies that enrolled participants with normal blood lipid profiles at baseline, those that included participants with high baseline BMI (≥30 kg/m^2^), or those used flaxseed and its derivatives as ALA source (Supplementary Table 4).

### Publication bias and sensitivity analysis

Funnel plots were generated for each outcome (CRP, TNF-α, IL-6, SBP, DBP, TG, TC, LDL cholesterol, and HDL cholesterol) (Supplementary Figures 5–13). According to Begg’s test, no publication bias was observed in the analysis of CRP (*P* = 0.30), TNF-α (*P* = 0.28), IL-6 (*P* = 0.39), SBP (*P* = 0.12), DBP (*P* = 0.92), TG (*P* = 0.15), TC (*P* = 0.24), and HDL cholesterol (*P* = 0.47), except for LDL cholesterol (*P* = 0.003). In addition, Egger’s regression test showed no publication bias for CRP (*P* = 0.19), TNF-α (*P* = 0.07), IL-6 (*P* = 0.61), SBP (*P* = 0.28), DBP (*P* = 0.83), TG (*P* = 0.15), TC (*P* = 0.31), and HDL cholesterol (*P* = 0.06). However, significant publication was found for TG (*P* = 0.005) and LDL cholesterol (*P* = 0.004), according to Egger’s regression test. Sensitivity analysis showed that the effect of dietary ALA supplementation on blood CRP, IL-6, TNF-α, SBP, DBP, TG, TC, LDL cholesterol, and HDL cholesterol was not changed by removing any 1 of the studies at a time (Data not shown).

## Discussion

This current systematic review and meta-analysis investigated the effect of dietary ALA on CVD risk factors, including inflammatory markers (CRP, IL-6, and TNF-α in serum), BP, and blood lipid profile (TG, TC, LDL cholesterol, and HDL cholesterol in serum) in patients with overweight or obesity. We found that supplementation with ALA was associated with a reduction in CRP, TNF-α, SBP, and TG concentrations. However, ALA intake was associated with increased LDL cholesterol. Meanwhile, there is no significant effect of ALA supplementation on IL-6, DBP, TC, and HDL cholesterol compared with the control.

We found that dietary ALA had a significant effect on decreasing the concentration of CRP and TNF-α, whereas it had no effect on IL-6. Subgroup analysis indicated that the anti-inflammatory effects of ALA supplementation were more prominent when intervention lasted ≥12 wk, used flaxseed or flaxseed oil as an ALA source at a dosage of ≥3 g/d, or included participants with higher baseline concentrations of CRP or TNF-α in serum. Similar to our findings, de Abreu et al. [28] demonstrated a significant decrease in serum CRP concentration in patients with chronic kidney disease after supplementation with ALA. In 2019, a systematic review and meta-analysis of 32 RCTs found that flaxseed-derived products had a significant effect on reducing CRP and TNF-α but not IL-6 in subjects with higher BMI (>30 kg/m^2^) in subgroup analysis. This is also in alignment with our results. When the serum concentration of inflammatory biomarkers is already at a low or normal range, there is no room for ALA supplementation to reduce it. The phenomenon was termed as “floor effect” by Nelson et al. [43]. Taken together, these results indicated that the effects of ALA on inflammatory markers might be more prominent in subjects of systematic inflammatory status.

Obesity induces persisting low-grade inflammation because of increasing macrophage infiltration, which further activates immune cells to release proinflammatory cytokines (i.e., CRP, TNF-α, and IL-6) into the circulation [62]. CRP is a validated and clinically useful gauge of the overall innate immune status of an individual in relation to CVD risk [63]. TNF-α and IL-6 are potent stimulants for CRP production. They are the most important cytokines released in large amounts during inflammatory response and play critical roles as mediators in CVD [64].

The mechanisms responsible for the anti-inflammatory benefit of ALA intake have not been clearly established, though several possible mechanisms have been proposed. First, ALA downregulates the gene expression of nitric oxide synthase, cyclooxygenase-2, and TNF-α via the inhibition of nuclear factor kappa B (NF-κB) and mitogen-activated protein kinase pathways [65]. In addition, ALA might improve the obese phenotype by reducing adipocyte hypertrophy, protein concentrations of inflammatory markers monocyte chemoattractant protein-1 and TNF-α, and T-cell infiltration in adipose tissue [66].

We found that ALA supplementation was associated with a significant reduction in SBP, whereas there was no significant alteration in DBP when compared to a control group. This is in line with the results of a meta-analysis investigating the effect of flaxseed-derived products and chia seeds [[67], [68], [69]]. However, walnut consumption did not significantly affect BP [70,71]. This suggests that the antihypertensive effect of ALA might be dependent on its food sources. A recent trial on 60 healthy Japanese adults showed that ALA from perilla frutescens leaf powder significantly decreased BP compared with the placebo group [72]. The effect of ALA on lowering BP might contribute to its inhibition of vascular endothelial activation and influence on the vascular endothelial beds.

In terms of blood lipid profile, we found a significant effect of ALA intake on reducing TG and no effect on HDL cholesterol, which was in agreement with a meta-analysis published in 2020 conducted by Yue et al. [27]. We also found that dietary intake of ALA significantly increased the concentration of LDL cholesterol in serum, which is the opposite of the result of Yue et al. [27]. However, considering the existing publication bias for TG and LDL cholesterol, these results should be interpreted with caution. If trials yielding null or unfavorable results had not been published as much as they should have, the present findings of the significant effect of ALA on decreasing TG and increasing LDL cholesterol could have been affected to some extent.

The well-established TG-lowering effect of fish oil, accompanied by an increase in LDL cholesterol, had been observed in adults with hypertriglyceridemia [73]. This might reveal a similar effect of dietary ALA on blood lipid profile.

Sodium-glucose cotransporter 2 (SGLT2) inhibition might play a part in the phenomenon of decreased TG and increased LDL cholesterol [74]. SGLT2 is a glucose transporter responsible for glucose renal reabsorption; its inhibition reduces plasma glucose concentrations [75]. Oral SGLT2 inhibitors, including canagliflozin and empagliflozin, have been widely used to treat Type 2 diabetes mellitus (T2DM). Meanwhile, these drugs have consistently been found to increase plasma LDL cholesterol concentrations. Intriguingly, despite increasing plasma LDL cholesterol, SGLT2 inhibitors are associated with reduced CVD, including heart failure and coronary disease [[76], [77], [78]]. Mouse studies suggested that increases in plasma LDL cholesterol following the use of SGLT2 inhibition were associated with delayed clearance of LDL from the circulation along with increased plasma lipoprotein lipase (LPL) activity. Decreases of angiopoietin-like protein (ANGPTL4) mRNA were observed in the heart, adipose, and skeletal muscle, allowing greater LPL activity [74]. ANGPTL4 is a circulating inhibitor of LPL expressed by cardiomyocytes [79].

Dietary ALA could upregulate ANGPTL4 expression mediated by peroxisome proliferator-activated receptor β/δ, which results in decreased cardiac uptake of plasma TG-derived fatty acids and decreased fatty acid-induced oxidative stress and lipid peroxidation [80]. Genetic studies have associated reduced ANGPTL4 with reduced CVD [81], suggesting that the clinical benefits of dietary ALA on CVD might be via SGLT2 inhibition.

To the best of the researcher’s knowledge, this study was the first comprehensive meta-analysis designed to analyze the effect of ALA supplementation on CVD risk factors in subjects with obesity or overweight. Our study has several strengths, including identifying randomly assigned trials with a detailed search strategy and subgroup analysis of various categories, including study duration, source, and dose of ALA, BMI of included participants, and baseline levels of interested outcomes. Our results regarding the blood lipid profile may also provide new insight into the mechanism of the cardioprotective effect of ALA. Limitations also need to be considered. We included trials with relatively small sample sizes (<100) and poor quality. The risk of bias mostly came from inadequate allocation, which might result in an inflated estimated effect size. Moreover, the included studies adopted different measurement techniques for each outcome. That variability might have potentially affected the effects. Adherence to the dietary intervention was predominantly self-reported, which might be prone to underreporting and random and systematic errors [52]. In addition, background diet might also affect how the subjects react to the ALA supplementation. Other factors, such as bioactive components contained in the supplementation, vitamin D, and gut microbiome status of the subjects, might also confound the effect of ALA supplementation [82,83]. Control groups were not consistent in all included trials. Heterogeneity exists in most of our analysis, which might be attributed to differences in the sources, doses, and duration of ALA intervention. Although we performed subgroup analysis on different doses of ALA, we were unable to analyze the optimal dose for a specific food source.

In conclusion, our meta-analysis demonstrated the beneficial effect of ALA supplementation on reducing CRP, TNF-α, SBP, and TG in individuals with obesity or overweight. However, we did not find any potential effect on IL-6, DBP, TC, and HDL cholesterol. Subgroup analyses implicated that dietary ALA supplementation at a dose of 3 g/d or higher from flaxseed and flaxseed oil has a more prominent effect on reducing serum CRP, serum TNF-α, SBP, and serum TG concentrations, particularly where intervention duration lasts ≥12 wk. Well-designed trials of large sample size, sufficient duration, and appropriate administration of ALA are needed in the future to optimize cardiovascular health in people with obesity or overweight.

## Author contributions

The authors’ responsibilities were as follows – SY: designed and conducted the research, performed the literature search, data extraction, and statistical analysis; wrote the initial draft and had primary responsibility for the final content; HX: performed literature search, data extraction, and quality assessment; JX: discussed the literature search results, performed quality assessment; JS and YW: created figures and tables; DX and YL: interpreted the data; WL and GS: revised the paper; and all authors: read and approved the final manuscript.

## Conflict of Interest

The authors report no conflicts of interest.

## Funding

This work was funded by the National Natural Science Foundation of China (Grant No. 82173509) and grants from the Chinese Nutrition Society Research Fund for Dietary Reference Intake (DRI).

## Appendix A Supplementary data

Multimedia component1 Multimedia component1
