# Supplementary material for: Effect of Alpha-Linolenic Acid Supplementation on Cardiovascular Disease Risk Profile in Individuals with Obesity or Overweight: A Systematic Review and Meta-Analysis of Randomized Controlled Trials
Source: Adv Nutr. 2023 Sep 29;14(6):1644–55. doi: 10.1016/j.advnut.2023.09.010 (PMC10721518; doi:10.1016/j.advnut.2023.09.010)
Supplement: Multimedia component1 [file mmc1.docx]

**Effect of alpha-linolenic acid supplementation on cardiovascular risk profile in individuals with obesity or overweight: A systematic review and meta-analysis of randomized controlled trials**

Shiyu Yin

Online Supplementary Material

**Supplementary Table S1.** PRISMA 2020 checklist

| **Section and Topic** | **Item #** | **Checklist item** | **Reported on page #** |
| --- | --- | --- | --- |
| **TITLE** | | |  |
| Title | 1 | Identify the report as a systematic review. | **1** |
| **ABSTRACT** | | |  |
| Abstract | 2 | See the PRISMA 2020 for Abstracts checklist. | **2** |
| **INTRODUCTION** | | |  |
| Rationale | 3 | Describe the rationale for the review in the context of existing knowledge. | **4-6** |
| Objectives | 4 | Provide an explicit statement of the objective(s) or question(s) the review addresses. | **6** |
| **METHODS** | | |  |
| Eligibility criteria | 5 | Specify the inclusion and exclusion criteria for the review and how studies were grouped for the syntheses. | **7** |
| Information sources | 6 | Specify all databases, registers, websites, organisations, reference lists and other sources searched or consulted to identify studies. Specify the date when each source was last searched or consulted. | **7** |
| Search strategy | 7 | Present the full search strategies for all databases, registers and websites, including any filters and limits used. | **7, Supplementary Table S2** |
| Selection process | 8 | Specify the methods used to decide whether a study met the inclusion criteria of the review, including how many reviewers screened each record and each report retrieved, whether they worked independently, and if applicable, details of automation tools used in the process. | **8** |
| Data collection process | 9 | Specify the methods used to collect data from reports, including how many reviewers collected data from each report, whether they worked independently, any processes for obtaining or confirming data from study investigators, and if applicable, details of automation tools used in the process. | **8** |
| Data items | 10a | List and define all outcomes for which data were sought. Specify whether all results that were compatible with each outcome domain in each study were sought (e.g. for all measures, time points, analyses), and if not, the methods used to decide which results to collect. | **8** |
|  | 10b | List and define all other variables for which data were sought (e.g. participant and intervention characteristics, funding sources). Describe any assumptions made about any missing or unclear information. | **8-9** |
| Study risk of bias assessment | 11 | Specify the methods used to assess risk of bias in the included studies, including details of the tool(s) used, how many reviewers assessed each study and whether they worked independently, and if applicable, details of automation tools used in the process. | **8** |
| Effect measures | 12 | Specify for each outcome the effect measure(s) (e.g. risk ratio, mean difference) used in the synthesis or presentation of results. | **9** |
| Synthesis methods | 13a | Describe the processes used to decide which studies were eligible for each synthesis (e.g. tabulating the study intervention characteristics and comparing against the planned groups for each synthesis (item #5)). | **8-9** |
|  | 13b | Describe any methods required to prepare the data for presentation or synthesis, such as handling of missing summary statistics, or data conversions. | **8-9** |
|  | 13c | Describe any methods used to tabulate or visually display results of individual studies and syntheses. | **8-9** |
|  | 13d | Describe any methods used to synthesize results and provide a rationale for the choice(s). If meta-analysis was performed, describe the model(s), method(s) to identify the presence and extent of statistical heterogeneity, and software package(s) used. | **9** |
|  | 13e | Describe any methods used to explore possible causes of heterogeneity among study results (e.g. subgroup analysis, meta-regression). | **9** |
|  | 13f | Describe any sensitivity analyses conducted to assess robustness of the synthesized results. | **9-10** |
| Reporting bias assessment | 14 | Describe any methods used to assess risk of bias due to missing results in a synthesis (arising from reporting biases). | **9-10** |
| Certainty assessment | 15 | Describe any methods used to assess certainty (or confidence) in the body of evidence for an outcome. | **/** |
| **RESULTS** | | |  |
| Study selection | 16a | Describe the results of the search and selection process, from the number of records identified in the search to the number of studies included in the review, ideally using a flow diagram. | **10, Figure 1** |
|  | 16b | Cite studies that might appear to meet the inclusion criteria, but which were excluded, and explain why they were excluded. | **10** |
| Study characteristics | 17 | Cite each included study and present its characteristics. | **10-13, Table 1** |
| Risk of bias in studies | 18 | Present assessments of risk of bias for each included study. | **14, Supplementary Table S3** |
| Results of individual studies | 19 | For all outcomes, present, for each study: (a) summary statistics for each group (where appropriate) and (b) an effect estimate and its precision (e.g. confidence/credible interval), ideally using structured tables or plots. | **14-17, Figure 2-6, Supplementary Figure S1-S4** |
| Results of syntheses | 20a | For each synthesis, briefly summarise the characteristics and risk of bias among contributing studies. | **14-18** |
|  | 20b | Present results of all statistical syntheses conducted. If meta-analysis was done, present for each the summary estimate and its precision (e.g. confidence/credible interval) and measures of statistical heterogeneity. If comparing groups, describe the direction of the effect. | **14-17, Supplementary Table S4** |
|  | 20c | Present results of all investigations of possible causes of heterogeneity among study results. | **14-17** |
|  | 20d | Present results of all sensitivity analyses conducted to assess the robustness of the synthesized results. | **14-17** |
| Reporting biases | 21 | Present assessments of risk of bias due to missing results (arising from reporting biases) for each synthesis assessed. | **17, Supplementary Figure S5-S13** |
| Certainty of evidence | 22 | Present assessments of certainty (or confidence) in the body of evidence for each outcome assessed. | **/** |
| **DISCUSSION** | | |  |
| Discussion | 23a | Provide a general interpretation of the results in the context of other evidence. | **18-21** |
|  | 23b | Discuss any limitations of the evidence included in the review. | **21** |
|  | 23c | Discuss any limitations of the review processes used. | **21** |
|  | 23d | Discuss implications of the results for practice, policy, and future research. | **21** |
| **OTHER INFORMATION** | | |  |
| Registration and protocol | 24a | Provide registration information for the review, including register name and registration number, or state that the review was not registered. | **6** |
|  | 24b | Indicate where the review protocol can be accessed, or state that a protocol was not prepared. | **6** |
|  | 24c | Describe and explain any amendments to information provided at registration or in the protocol. | **/** |
| Support | 25 | Describe sources of financial or non-financial support for the review, and the role of the funders or sponsors in the review. | **1** |
| Competing interests | 26 | Declare any competing interests of review authors. | **/** |
| Availability of data, code and other materials | 27 | Report which of the following are publicly available and where they can be found: template data collection forms; data extracted from included studies; data used for all analyses; analytic code; any other materials used in the review. | **/** |

*From:*  Page MJ, McKenzie JE, Bossuyt PM, Boutron I, Hoffmann TC, Mulrow CD, et al. The PRISMA 2020 statement: an updated guideline for reporting systematic reviews. BMJ 2021;372:n71. doi: 10.1136/bmj.n71

**Supplementary Table S2.** MesH and non-MesH search terms

| **Non-MeSH terms** | **MeSH terms** |
| --- | --- |
| “alpha-linolenic acid” OR “alpha linolenic acid” OR “α-linolenic acid” OR “α linolenic acid” OR “Linolenate” OR “Linolenic Acid” OR “Unsaturated Fatty Acid” OR “Unsaturated Fatty Acids” OR “Polyunsaturated Fatty Acid” OR “Polyunsaturated Fatty Acids” OR “Omega 3 fatty acid” OR “Omega 3 fatty acids” OR “Omega-3 fatty acid” OR “Omega-3 fatty acids” OR “n-3 Fatty Acids” OR “n-3 Fatty Acid” OR “Ome ga 3” OR “Omega-3” OR “n-3 Oils” OR “n-3 Oil” OR “walnut” OR “flaxseed” OR “canola” OR “rapeseed” OR “chia seed” OR “perilla seed oil” OR “flax” OR “Linum usitatissimum” OR “secoisolariciresinol diglucoside” OR “lignan” OR “linseed” OR “SDG” OR “ALA” | “obesity” OR “overweight” OR “obese” |

**Supplementary Table S3.** Assessment of risk of bias for the included studies using Cochrane criteria**.**

| Study | Random sequence generation | Allocation concealment | Blinding of participants and personnel | Blinding of outcome assessments | Incomplete outcome data | Selective outcome reporting | Other potential threats to validity | Overall quality |
| --- | --- | --- | --- | --- | --- | --- | --- | --- |
| Ahmadniay, 2021 | L | L | L | L | L | L | L | G |
| Baxheinrich, 2012 | U | U | U | U | L | L | L | F |
| de Oliveira, 2017 | U | U | L | L | U | U | L | F |
| Egert, 2014 | U | U | U | U | L | L | L | F |
| Eriksen, 2020 | L | H | H | L | L | U | L | P |
| Faintuch, 2011 | U | H | H | U | L | L | L | P |
| Hajiahmadi, 2020 | U | U | U | L | L | L | L | F |
| Hutchins, 2013 | U | L | H | L | L | L | L | P |
| Hwang, 2019 | L | H | H | U | L | L | L | P |
| Jamilian, 2019 | L | L | L | L | L | L | L | G |
| Jamka, 2021 | L | L | L | L | L | L | L | G |
| Joris, 2020 | L | U | U | U | L | L | L | F |
| Katz, 2012 | L | H | H | U | L | U | L | P |
| Kruse, 2020 | L | H | H | H | L | L | L | P |
| Machado, 2015 | L | H | H | L | L | L | L | P |
| Ndanuko, 2018 | L | L | L | L | L | U | L | F |
| Nelson, 2007 | L | U | H | U | U | L | L | P |
| Nieman, 2012 | U | L | L | L | L | L | L | F |
| Rock, 2017 | L | L | L | L | L | U | L | G |
| Shareghfarid, 2022 | L | L | L | L | L | L | L | G |

F, fair quality; G, good quality; H, high risk of bias; L, low risk of bias; P, poor quality; U, unclear risk of bias.

**Supplementary Table S4**. Subgroup analysis of the effect of dietary alpha-linolenic acid (ALA) supplementation on inflammatory biomarkers

|  | **Subgroup** | **No. of effect size** | **SMD (95% CI)** | **P-value** | **I^2^ (%)** | **P for heterogeneity** |
| --- | --- | --- | --- | --- | --- | --- |
| **Inflammatory markers** | | | | | | |
| **CRP**  (mg/l) | **Total** | 12 | **-0.38 (-0.72, -0.04)** | **0.03** | 64.20 | < 0.001 |
|  | **Study duration (wk)** |  |  |  |  |  |
|  | < 12 weeks | 2 | -1.13 (-3.74, 1.48) | 0.08 | 95.09 | < 0.001 |
|  | ≥ 12 weeks | 10 | -0.265 (-0.48, -0.056) | **0.02** | 9.559 | 0.35 |
|  | **Baseline CRP level** |  |  |  |  |  |
|  | Normal (<3.0 mg/L) | 4 | -0.075 (-0.42, 0.27) | 0.37 | 0 | 0.53 |
|  | Elevated (≥ 3.0 mg/L) | 8 | -0.57 (-1.04, -0.09) | **0.02** | 73.43 | < 0.001 |
|  | **Baseline BMI** |  |  |  |  |  |
|  | < 30 (kg/m^2^) | 6 | -0.18 (-1.44, 0.087) | 0.09 | 21.57 | 0.27 |
|  | ≥ 30 (kg/m^2^) | 6 | -0.68 (-1.32, -0.03) | **0.04** | 78.05 | < 0.001 |
|  | **ALA supplementation source** |  |  |  |  |  |
|  | Flaxseed and its derivatives | 7 | -0.27 (-0.52, -0.019) | **0.035** | 44.24 | 0.096 |
|  | Walnut | 0 | - | **-** | - | - |
|  | Other | 4 | -0.66 (-1.67, 0.35) | 0.20 | 84.90 | < 0.001 |
|  | **ALA dose (g/d)** |  |  |  |  |  |
|  | < 3g | 1 | -0.31 (-0.93, 0.32) | 0.34 | - | - |
|  | ≥ 3g | 11 | -0.40 (-0.77, -0.02) | **0.04** | 67.46 | 0.001 |
|  | **Study quality** |  |  |  |  |  |
|  | Good | 1 | -0.31 (-0.93, 0.32) | 0.34 | - | - |
|  | Fair | 5 | -0.13 (-0.39, 0.13) | 0.34 | 0 | 0.84 |
|  | Poor | 6 | -0.68 (-1.44, 0.09) | 0.08 | 80.42 | 0 |
| **IL-6**  (pg/ml) | **Total** | 8 | 0.12 (-0.14, 0.40) | 0.33 | 21.87 | 0.26 |
|  | **Study duration (wk)** |  |  |  |  |  |
|  | < 12 weeks | 1 | -0.18 (-0.73, 0.37) | 0.52 | - | - |
|  | ≥ 12 weeks | 7 | 0.18 (-0.067, 0.15) | 0.44 | 20.67 | 0.27 |
|  | **Baseline CRP level** |  |  |  |  |  |
|  | Normal (<3.0 mg/L) | 2 | -0.16 (-0.172, 1.40) | 0.77 | 72.59 | 0.057 |
|  | Elevated (≥ 3.0 mg/L) | 6 | 0.14 (-0.096, 0.38) | 0.24 | 0 | 0.42 |
|  | **Baseline BMI** |  |  |  |  |  |
|  | < 30 (kg/m^2^) | 5 | 0.026 (-0.26, 0.32) | 0.86 | 17.37 | 0.30 |
|  | ≥ 30 (kg/m^2^) | 3 | 0.28 (-0.094, 0.64) | 0.15 | 34.25 | 0.22 |
|  | **ALA supplementation source** |  |  |  |  |  |
|  | Flaxseed and its derivatives | 5 | 0.026 (-0.26, 0.32) | 0.86 | 17.37 | 0.30 |
|  | Walnut | 0 | - | - | - | - |
|  | Other | 3 | 0.28 (-0.094, 0.64) | 0.15 | 32.25 | 0.22 |
|  | **ALA dose (g/d)** |  |  |  |  |  |
|  | < 3g | 0 | - | - | - | - |
|  | ≥ 3g | 8 | -0.36 (-1.26, 0.54) | 0.48 | 83.08 | 0.003 |
|  | **Study quality** |  |  |  |  |  |
|  | Good | 0 | - | - | - | - |
|  | Fair | 4 | 0.21 (-0.086, 0.51) | 0.17 | 10.55 | 0.34 |
|  | Poor | 4 | -0.02 (-0.50, 0.46) | 0.93 | 36.66 | 0.19 |
| **TNF-α**  (pg/ml) | **Total** | 9 | -0.45 (-0.72, -0.17) | **0.001** | 33.45 | 0.15 |
|  | **Study duration (wk)** |  |  |  |  |  |
|  | < 12 weeks | 1 | - | - | - | - |
|  | ≥ 12 weeks | 8 | -0.41(-0.64, -0.18) | **0.001** | 41.39 | 0.10 |
|  | **Baseline TNF-α level** |  |  |  |  |  |
|  | Normal (<2.2 pg/ml) | 3 | -0.11 (-0.39, 0.18) | 0.46 | 0 | 0.64 |
|  | Elevated (≥ 2.2 pg/ml) | 6 | -0.78 (-1.11, -0.45) | **< 0.001** | 0 | 0.91 |
|  | **Baseline BMI** |  |  |  |  |  |
|  | < 30 (kg/m^2^) | 6 | -0.43 (-0.69, -0.17) | **0.01** | 27.26 | 0.23 |
|  | ≥ 30 (kg/m^2^) | 3 | -0.53 (-1.22, 0.16) | 0.13 | 59.17 | 0.086 |
|  | **ALA supplementation source** |  |  |  |  |  |
|  | Flaxseed and its derivatives | 5 | -0.61 (-0.92, -0.30) | **< 0.001** | 0 | 0.66 |
|  | Walnut | 1 | - | **-** | - | - |
|  | Other | 3 | -0.53 (-1.22, 0.16) | 0.13 | 59.16 | 0.086 |
|  | **ALA dose (g/d)** |  |  |  |  |  |
|  | < 3g | 0 | - | - | - | - |
|  | ≥ 3g | 9 | -0.45 (-0.72, -0.17) | **0.001** | 33.35 | 0.15 |
|  | **Study quality** |  |  |  |  |  |
|  | Good | 1 | - | - | - | - |
|  | Fair | 5 | -0.50 (-0.78, -0.23) | **0.01** | 49.98 | 0.09 |
|  | Poor | 3 | -0.46 (-0.92, -0.03) | 0.05 | 0 | 0.72 |
| **Blood pressure** | | | | | | |
| **SBP**  (mm Hg) | **Total** | 11 | -0.37 (-0.66, -0.08) | **< 0.001** | 71.58 | < 0.001 |
|  | **Study duration (wk)** |  |  |  |  |  |
|  | < 12 weeks | 2 | -0.21 (-0.56, 0.15) | 0.25 | 0 | 0.54 |
|  | ≥ 12 weeks | 9 | -0.41 (-0.77, -0.05) | **0.03** | 76.78 | < 0.001 |
|  | **Baseline BP** |  |  |  |  |  |
|  | Normal | 2 | -0.50 (-1.85, 0.85) | 0.47 | 94.98 | < 0.001 |
|  | Elevated (SBP ≥ 130 or DBP ≥ 80) | 9 | -0.24 (-0.41, -0.07) | **0.01** | 38.14 | 0.11 |
|  | **Baseline BMI** |  |  |  |  |  |
|  | < 30 (kg/m^2^) | 3 | -0.58 (-1.42, 0.27) | 0.18 | 90.20 | < 0.001 |
|  | ≥ 30 (kg/m^2^) | 8 | -0.18 (-0.36, -0.001） | **0.049** | 24.34 | 0.24 |
|  | **ALA supplementation source** |  |  |  |  |  |
|  | Flaxseed and its derivatives | 3 | -0.34 (-0.66, -0.03) | **0.03** | 37.11 | 0.20 |
|  | Walnut | 4 | -0.33 (-0.95, 0.28) | 0.29 | 87.97 | < 0.001 |
|  | Other | 4 | -0.42 (-0.91, 0.06) | **0.04** | 57.09 | 0.07 |
|  | **ALA dose (g/d)** |  |  |  |  |  |
|  | < 3g | 2 | -0.17 (-0.48, 0.15) | 0.30 | 0 | 0.78 |
|  | ≥ 3g | 9 | -0.43 (-0.81, 0.06) | **0.02** | 76.58 | < 0.001 |
|  | **Study quality** |  |  |  |  |  |
|  | Good | 1 | -1.19 (-1.62, -0.77) | **-** | - | - |
|  | Fair | 6 | -0.37 (-0.71, -0.02) | **0.04** | 59.87 | 0.03 |
|  | Poor | 4 | -0.06 (-0.32, 0.21) | 0.67 | 0 | 0.51 |
| **DBP**  (mm Hg) | **Total** | 9 | -0.32 (-0.74, 0.10) | 0.14 | 85.85 | < 0.001 |
|  | **Study duration (wk)** |  |  |  |  |  |
|  | < 12 weeks | 2 | -0.75 (-1.77, 0.28) | 0.16 | 86.42 | 0.01 |
|  | ≥ 12 weeks | 7 | -0.20 (-0.66, 0.260) | 0.40 | 85.55 | < 0.001 |
|  | **Baseline BP** |  |  |  |  |  |
|  | Normal | 2 | -0.15 (-1.52, 1.22) | 0.83 | 95.23 | < 0.001 |
|  | Elevated | 7 | -0.37 (-0.82, 0.07) | 0.10 | 82.44 | < 0.001 |
|  | **Baseline BMI** |  |  |  |  |  |
|  | < 30 (kg/m^2^) | 3 | -0.36 (-1.28, 0.57) | 0.45 | 91.90 | < 0.001 |
|  | ≥ 30 (kg/m^2^) | 6 | -0.30 (-0.81, 0.21) | 0.25 | 84.29 | < 0.001 |
|  | **ALA supplementation source** |  |  |  |  |  |
|  | Flaxseed and its derivatives | 3 | -0.37 (-1.50, 0.76) | 0.52 | 89.96 | < 0.001 |
|  | Walnut | 4 | -0.11 (-0.69, 0.48) | 0.72 | 86.68 | < 0.001 |
|  | Other | 2 | -0.57 (-0.89, -0.25) | **< 0.001** | 0 | 0.65 |
|  | **ALA dose (g/d)** |  |  |  |  |  |
|  | < 3g | 1 | -1.26 (-1.74, -0.77) | **< 0.001** | - | - |
|  | ≥ 3g | 8 | -0.20 (-0.61, 0.21) | 0.33 | 83.15 | < 0.001 |
|  | **Study quality** |  |  |  |  |  |
|  | Good | 1 | -0.85 (-1.26, -0.44) | - | - | - |
|  | Fair | 4 | -0.44 (-0.85, -0.02) | **0.04** | 72.72 | 0.01 |
|  | Poor | 4 | 0.03 (-0.98, 1.03) | 0.96 | 91.96 | < 0.001 |
| Blood lipid | | | | | | |
| **TG**  (mg/dL) | **Total** | 10 | -4.41 (-5.99, -2.82) | **< 0.001** | 97.24 | < 0.001 |
|  | **Study duration (wk)** |  |  |  |  |  |
|  | < 12 weeks | 2 | -11.16 (-32.76, 10.45) | 0.31 | 99.32 | < 0.001 |
|  | ≥ 12 weeks | 8 | -2.72 (-4.21, -1.23) | **< 0.001** | 96.05 | < 0.001 |
|  | **Baseline blood lipid** |  |  |  |  |  |
|  | Normal | 5 | -9.99 (13.92, -6.05) | **< 0.001** | 97.90 | < 0.001 |
|  | Elevated | 5 | -2.19 (-3.93, -0.45) | **0.01** | 96.84 | < 0.001 |
|  | **Baseline BMI** |  |  |  |  |  |
|  | < 30 (kg/m^2^) | 6 | -4.96 (-7.11, -2.81) | **< 0.001** | 96.99 | < 0.001 |
|  | ≥ 30 (kg/m^2^) | 4 | -4.37 (-7.45, -1.29) | **0.01** | 98.12 | < 0.001 |
|  | **ALA supplementation source** |  |  |  |  |  |
|  | Flaxseed and its derivatives | 6 | -13.57 (-19.40, -7.74) | **< 0.001** | 98.39 | < 0.001 |
|  | Walnut | 4 | -0.28 (-0.64, 0.09) | 0.14 | 52.69 | 0.096 |
|  | Other | 0 | - | - | - | - |
|  | **ALA dose (g/d)** |  |  |  |  |  |
|  | < 3g | 2 | -11.35 (-32.57, 9.86) | 0.29 | 99.29 | < 0.001 |
|  | ≥ 3g | 8 | -2.60 (-4.06, -1.14) | **< 0.001** | 96.04 | < 0.001 |
|  | **Study quality** |  |  |  |  |  |
|  | Good | 3 | -0.36 (-0.93, 0.21) | 0.22 | 71.73 | 0.03 |
|  | Fair | 1 | -19.07 (-22.55, -15.59) | **-** | - | - |
|  | Poor | 6 | -6.69 (-9.68, -3.69) | **< 0.001** | 97.58 | < 0.001 |
| **TC** (mg/dL) | **Total** | ` | 0.60 (-0.33, 1.53) | 0.16 | 95.16 | < 0.001 |
|  | **Study duration (wk)** |  |  |  |  |  |
|  | < 12 weeks | 2 | 1.76 (-1.77, 5.300 | 0.33 | 98.31 | < 0.001 |
|  | ≥ 12 weeks | 10 | 0.26 (-0.65, 1.16) | 0.58 | 93.33 | < 0.001 |
|  | **Baseline blood lipid** |  |  |  |  |  |
|  | Normal | 5 | 0.72 (-0.99, 2.43) | 0.41 | 96.17 | < 0.001 |
|  | Elevated | 7 | 0.49 (-0.73, 1.71) | 0.43 | 94.73 | < 0.001 |
|  | **Baseline BMI** |  |  |  |  |  |
|  | < 30 (kg/m^2^) | 6 | 0.24 (-0.72, 1.21) | 0.62 | 92.70 | < 0.001 |
|  | ≥ 30 (kg/m^2^) | 6 | 1.46 (-0.50, 3.42) | 0.14 | 96.75 | < 0.001 |
|  | **ALA supplementation source** |  |  |  |  |  |
|  | Flaxseed and its derivatives | 6 | 1.05 (-0.53, 2.62) | 0.20 | 95.52 | < 0.001 |
|  | Walnut | 4 | -0.28 (-0.52, -0.04) | **0.03** | 3.806 | 0.37 |
|  | Other | 2 | 6.72 (-13.20, 26.64) | 0.51 | 98.16 | < 0.001 |
|  | **ALA dose (g/d)** |  |  |  |  |  |
|  | < 3g | 2 | 1.58 (-2.31, 5.48) | 0.43 | 98.51 | < 0.001 |
|  | ≥ 3g | 10 | 0.29 (-0.60, 1.19) | 0.52 | 93.27 | < 0.001 |
|  | **Study quality** |  |  |  |  |  |
|  | Good | 3 | -0.49 (-0.77, -0.20) | **0.001** | 0 | 0.95 |
|  | Fair | 3 | 4.35 (-1.31, 10.02） | 0.13 | 97.63 | < 0.001 |
|  | Poor | 6 | 0.74 (-0.63, 2.11) | 0.29 | 94.98 | < 0.001 |
| **LDL-C**  (mg/dL) | **Total** | 10 | 1.32 (0.05, 2.59) | **0.04** | 96.76 | < 0.001 |
|  | **Study duration (wk)** |  |  |  |  |  |
|  | < 12 weeks | 2 | 51.69 (-50.19, 153.57) | 0.32 | 99.36 | < 0.001 |
|  | ≥ 12 weeks | 8 | 0.71 (-0.28, 1.70) | 0.16 | 949.21 | < 0.001 |
|  | **Baseline blood lipid** |  |  |  |  |  |
|  | Normal | 5 | 4.30 (1.28, 7.32) | **0.01** | 97.76 | < 0.001 |
|  | Elevated | 5 | 0.50 (-0.85, 1.86) | 0.47 | 95.91 | < 0.001 |
|  | **Baseline BMI** |  |  |  |  |  |
|  | < 30 (kg/m^2^) | 6 | 1.05 (-0.31, 2.41) | 0.13 | 95.86 | < 0.001 |
|  | ≥ 30 (kg/m^2^) | 4 | 3.70 (0.43, 6.960 | **0.03** | 98.08 | < 0.001 |
|  | **ALA supplementation source** |  |  |  |  |  |
|  | Flaxseed and its derivatives | 6 | 4.91 (1.85, 7.97) | **0.002** | 97.78 | < 0.001 |
|  | Walnut | 4 | -0.44 (-0.91, 0.04) | 0.07 | 71.95 | 0.01 |
|  | Other | 0 | - | - | - | - |
|  | **ALA dose (g/d)** |  |  |  |  |  |
|  | < 3g | 2 | 53.55 (-50.59, 153.69) | 0.32 | 99.36 | < 0.001 |
|  | ≥ 3g | 8 | 0.74 (-0.24, 1.72) | 0.14 | 94.22 | < 0.001 |
|  | **Study quality** |  |  |  |  |  |
|  | Good | 3 | -0.46 (-1.13, 0.19) | 0.17 | 79.04 | 0.01 |
|  | Fair | 1 | 3.71 (2.87, 4.55) | **-** | - | - |
|  | Poor | 6 | 2.285 (0.54, 5.17) | **0.02** | 97.20 | < 0.001 |
| **HDL-C**  (mg/dL) | **Total** | 10 | -0.50 (-1.45, 0.45) | 0.30 | 95.03 | < 0.001 |
|  | **Study duration (wk)** |  |  |  |  |  |
|  | < 12 weeks | 2 | -53.19 (-158.14, 51.76) | 0.32 | 99.36 | < 0.001 |
|  | ≥ 12 weeks | 8 | 0.35 (-0.10, 1.09) | 0.36 | 90.26 | < 0.001 |
|  | **Baseline blood lipid** |  |  |  |  |  |
|  | Normal | 5 | -3.15 (-6.28, -0.03) | **0.04** | 97.82 | < 0.001 |
|  | Elevated | 4 | 0.53 (-0.33, 1.35) | 0.23 | 90.42 | < 0.001 |
|  | **Baseline BMI** |  |  |  |  |  |
|  | < 30 (kg/m^2^) | 6 | 0.44 (-0.56, 1.44) | 0.39 | 92.92 | < 0.001 |
|  | ≥ 30 (kg/m^2^) | 4 | -3.56 (-3.84, -0.29) | **0.03** | 98.08 | < 0.001 |
|  | **ALA supplementation source** |  |  |  |  |  |
|  | Flaxseed and its derivatives | 6 | -2.1 (-4.98, 0.77) | 0.15 | 97.71 | < 0.001 |
|  | Walnut | 4 | -0.002 (-0.24, 0.24) | 0.99 | 0 | 0.68 |
|  | Other | 0 | - | - | - | - |
|  | **ALA dose (g/d)** |  |  |  |  |  |
|  | < 3g | 2 | -53.36 (-157.97, 51.25) | 0.32 | 99.35 | < 0.001 |
|  | ≥ 3g | 8 | 0.39 9-0.33, 1.12) | 0.29 | 89.87 | < 0.001 |
|  | **Study quality** |  |  |  |  |  |
|  | Good | 3 | -0.08 (-0.37, 0.20) | 0.57 | 0 | 0.67 |
|  | Fair | 1 | 1.09 (0.55, 1.64) | - | - | - |
|  | Poor | 6 | -2.48 (-4.58, -0.39) | **0.02** | 96.99 | < 0.001 |

**Supplementary Figure S1**. Forest plot of standard mean difference (SDM) and 95% confidence interval (CI) for the effect of dietary ALA supplementation on circulating triglyceride (TG) in people with obesity or overweight

**Supplementary Figure S2**. Forest plot of standard mean difference (SDM) and 95% confidence interval (CI) for the effect of dietary ALA supplementation on circulating total cholesterol (TC) in people with obesity or overweight

**Supplementary Figure S3**. Forest plot of standard mean difference (SDM) and 95% confidence interval (CI) for the effect of dietary ALA supplementation on circulating low-density lipoprotein cholesterol (LDL-C) in people with obesity or overweight

**Supplementary Figure S4**. Forest plot of standard mean difference (SDM) and 95% confidence interval (CI) for the effect of dietary ALA supplementation on circulating high-density lipoprotein cholesterol (HDL-C) in people with obesity or overweight

**Supplementary Figure S5**. Funnel plot representing publication bias in the studies reporting the effect of ALA on effect of dietary ALA supplementation on CRP in people with obesity or overweight

**Supplementary Figure S6**. Funnel plot representing publication bias in the studies reporting the effect of ALA on effect of dietary ALA supplementation on TNF in people with obesity or overweight

**Supplementary Figure S7**. Funnel plot representing publication bias in the studies reporting the effect of ALA on effect of dietary ALA supplementation on IL-6 in people with obesity or overweight

**Supplementary Figure S8**. Funnel plot representing publication bias in the studies reporting the effect of ALA on effect of dietary ALA supplementation on systolic blood pressure (SBP) in people with obesity or overweight

**Supplementary Figure S9**. Funnel plot representing publication bias in the studies reporting the effect of ALA on effect of dietary ALA supplementation on diastolic blood pressure (DBP) in people with obesity or overweight

**Supplementary Figure S10**. Funnel plot representing publication bias in the studies reporting the effect of ALA on effect of dietary ALA supplementation on TG in people with obesity or overweight

**Supplementary Figure S11**. Funnel plot representing publication bias in the studies reporting the effect of ALA on effect of dietary ALA supplementation on TC in people with obesity or overweight

**Supplementary Figure S12**. Funnel plot representing publication bias in the studies reporting the effect of ALA on effect of dietary ALA supplementation on LDL-C in people with obesity or overweight

**Supplementary Figure S13**. Funnel plot representing publication bias in the studies reporting the effect of ALA on effect of dietary ALA supplementation on HDL-C in people with obesity or overweight
